# Cooperativity, absolute interaction, and algebraic optimization

**DOI:** 10.1007/s00285-020-01540-8

**Published:** 2020-09-23

**Authors:** Nidhi Kaihnsa, Yue Ren, Mohab Safey El Din, Johannes W. R. Martini

**Affiliations:** 1 Division of Applied Mathematics, Brown University, 182 George Street, Providence, RI 02912 USA; 2 Department of Mathematics, Swansea University, Swansea University Bay Campus, Swansea, SA1 8EN UK; 3 Laboratoire d’Informatique de Paris 6, LIP6, Équipe PolSys, Sorbonne Université, CNRS, Paris, France; 4 International Maize and Wheat Improvement Center, Texcoco, Mexico

**Keywords:** 92C40, 90C23

## Abstract

We consider a measure of cooperativity based on the minimal interaction required to generate an observed titration behavior. We describe the corresponding algebraic optimization problem and show how it can be solved using the nonlinear algebra tool SCIP. Moreover, we compute the minimal interactions and minimal molecules for several binding polynomials that describe the oxygen binding of various hemoglobins under different conditions. We compare their minimal interaction with the maximal slope of the Hill plot, and discuss similarities and discrepancies with a view towards the shapes of the binding curves.

## Introduction

**Fig. 1:**
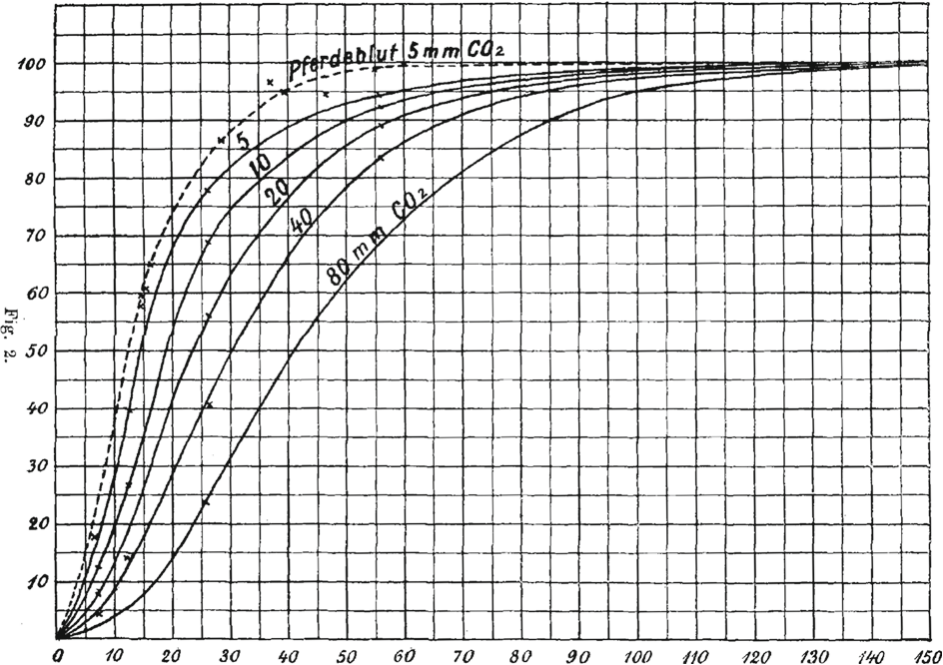
The oxygen concentration in dog and horse blood in relation to the ambient oxygen concentration (Bohr et al. 1904, Fig. 2)

Interaction between components is a fundamental feature of biological systems. While a simple system of independent subunits is completely defined by its subunits, a complex system with interactions is more than the sum of its parts. A classical example of a small biological system with non-trivial interaction is hemoglobin with its four binding sites for oxygen (Bohr et al. 1904; Barcroft 1913; Hill 1913). The *ligand* oxygen binds to the four binding sites of the *(target) molecule* hemoglobin and the interaction can be seen on the *overall (isotherm) binding curve*, which relates the average ligand saturation inside the system to the ligand concentration outside the system at constant temperature (Hill 1985). While the binding curve of an independent system is linear, the binding curve of hemoglobin has a sigmoidal shape (Barcroft 1913; Hill 1913), see Fig. 1.

The sigmoidal shape implies that the extremal states of full and zero saturation are more stable than the intermediate states of partial saturation. This phenomenon is commonly referred to as *cooperativity*, named after the intuition that bound ligands affect, either positively or negatively, the chances of new ligands binding to the still open sites (see Stefan and Le Novère 2013 for a review and interpretations). Cooperativity is ubiquitous in nature. It is an essential trait for transport molecules, and its importance in the formation of multi-protein complexes (Roy et al. 2017), in general signal transduction processes (Salakhieva et al. 2016; Lenaerts et al. 2009; Martini et al. 2016b) and in the regulation of noise (Gutierrez et al. 2009; Monteoliva et al. 2013) has been discussed.

When investigating cooperativity, one interesting problem is to connect macroscopic behavior to microscopic behavior. Here, macroscopic refers to the measured average saturation at varying ligand concentration, and microscopic refers to which individual sites are saturated and how the different sites interact. An example is the aforementioned interpretation of occupied sites increasing the probability of ligands binding to unoccupied sites (Stefan and Le Novère 2013; Onufriev and Ullmann 2004). Ideally, such connections between microscopic and macroscopic behavior provide insight on the potential structure and the inner workings of the molecule.

However, the overall binding curve alone does not allow for much inference on the individual site binding curves, similar to how a large sum allows for little inference on its summands. Given a fixed overall binding curve, there generally is no unique molecule, defined by binding and interaction energies, with the prescribed macroscopic binding behavior, but infinitely many. Thus many works feature additional constraints which reduce the ambiguity, such as considering only small system with one or two binding sites (Hunter and Anderson 2009) or symmetric systems with identical binding sites (Abeliovich 2016; Rong et al. 2019). These constraints have the added benefit of simplifying the mathematical problem, allowing for easier inference on interaction energy terms, conditional probabilities, correlations or other quantities. Ideally, constraints should be motivated by knowledge on the chemical structure of the molecule.

A biologically and mathematically interesting question is for instance whether there exist a molecule with independent sites for a fixed overall binding curve. Assuming evolutionary pressure on the transport properties of hemoglobin, it should not be possible to model the system’s overall binding curve through an independent system, since otherwise the tetrameric structure of hemoglobin would hold no advantage over four monomers.

The decoupled sites representation (Onufriev et al. 2001; Martini and Ullmann 2013) is a central theorem in this context, which states that every overall binding curve can be generated by a unique *hypothetical molecule* with possibly non-real binding energies and independent binding sites. The binding energies are determined by the roots of the *binding polynomial* of the overall binding curve. In particular, the realness of the roots show whether interaction is necessary to achieve the overall binding behavior and thus provide a qualitative definition of cooperativity.

In case interaction is required to generate the overall binding curve, a natural question is how to quantify the degree of cooperativity. This is classically done using the maximal slope of the Hill plot, which is the plot of binding curve on a logarithmic x-axis (see Fig. 6). A new approach (Martini 2017) is to look at the minimal interaction required to generate the considered overall binding curve. The minimal interaction quantifies the minimal deviation from an independent system that is necessary to explain the observed overall binding behavior.

The manuscript in hand studies the problem of finding molecules generating a given overall binding curve with minimal interaction. We show that looking for the minimal interaction energy required to generate a given overall titration behavior, leads to an algebraic optimization problem which can be partially tackled using software such as SCIP (Gleixner et al. 2018). We calculate the minimal molecules of various binding curves in existing datasets (Connelly et al. 1986; Ikeda-Saito et al. 1983; Imai 1973). These binding curves describe the oxygen binding of hemoglobins under different chemical treatments and under different temperatures. We compare their minimal interaction with the maximal slope of the Hill plot, and discuss similarities and discrepancies with a view towards the shapes of the binding curves.

## Recapitulation of the mathematical framework

In this section, we briefly review the model for ligand binding based on the grand canonical ensemble of statistical mechanics and recall the notion of minimal absolute interaction from (Martini 2017). We refer the reader to (Ben-Naim 2013; Wyman and Gill 1990; Hill 1985) for detailed expositions on the model.

### Definition 2.1

A *hypothetical molecule* $\mathrm{W}$ with *n* sites is a complex point, whose coordinates are indexed by the subsets of $[n]:=\{1,\ldots ,n\}$ and with $w_\emptyset =1$:

$$\begin{aligned} \mathrm{W}= (w_I)_{I\subseteq [n]} \in \mathbb {\mathbb {C}}^{2^n}. \end{aligned}$$

A *(real) molecule* is a hypothetical molecule whose coordinates are real and positive.

**Fig. 2:**
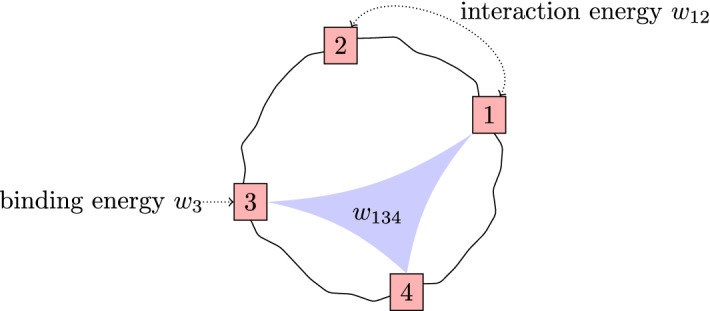
A molecule with 4 binding sites

We refer to $w_I$ as *binding energies* if $|I|=1$ and *interaction energies* if $|I|>1$. For the sake of brevity, we abbreviate $w_{\{i_1,\ldots ,i_r\}}$ to $w_{i_1\ldots i_r}$ for $i_1<\dots <i_r$ (see Fig. 2).

Note that we refer to $w_{i_1 \dots i_r}$ as *energy* even though it is actually representing the exponential of an energy difference (Ben-Naim 2013; Wyman and Gill 1990; Schellman 1975). To be precise:

- The binding energy $w_i$ encodes the difference between the free energies of the completely unsaturated state and the state with a single ligand bound to site *i*.
- The interaction energy $w_{i_1 i_2}$ encodes the difference between the free energy when sites $i_1,i_2$ are occupied and the sum of the energies when either $i_1$ or $i_2$ is occupied.
- Analogously, $w_{i_1 \dots i_r}$ encodes the difference between the free energy when sites $i_1,\dots ,i_r$ are occupied and the sum of all energies when a proper subset of $i_1,\dots ,i_r$ are occupied.

As such, the only “sensible” values for $w_{i_1 \dots i_r}$ are positive real numbers. However, mathematically it is easier to work with hypothetical molecules, and they allow for interesting phenomena such as the decoupled sites representation (Onufriev et al. 2001; Martini and Ullmann 2013). In this article, we will mainly focus on real molecules.

### Definition 2.2

The *overall binding curve* of a real molecule $\mathrm{W}=(w_I)_{I\subseteq [n]}\in ({\mathbb {R}}_{>0})^{2^n}$ with *n* sites is defined as

$$\begin{aligned} \Psi (\Lambda ) = \frac{ n a_n \Lambda ^n + (n-1)a_{n-1} \Lambda ^{n-1} + \dots + a_1 }{ a_n \Lambda ^n + a_{n-1} \Lambda ^{n-1} + \dots + a_1 + a_0}, \end{aligned}$$

also commonly known as the Adair equation (Adair et al. 1925; Stefan and Le Novère 2013), with positive real coefficients

$$\begin{aligned} a_k := \sum _{|I|=k} \prod _{I'\subseteq I} w_{I'} \in {\mathbb {R}}_{>0}. \end{aligned}$$

The *binding polynomial* $\Phi (\mathrm{W})$ is the univariate polynomial of degree *n* in its denominator,

$$\begin{aligned} \Phi (\mathrm{W}):=a_n \Lambda ^n + \cdots + a_1 \Lambda + a_0. \end{aligned}$$

The binding polynomial $\Phi$ uniquely determines the overall binding curve $\Psi$.

### Example 2.3

Let $\mathrm{W}=(w_I)_{I\subseteq [3]}\in ({\mathbb {R}}_{>0})^{2^3}$ be a molecule with 3 sites. The binding polynomial $\Phi (\mathrm{W})=a_3\Lambda ^3+a_2\Lambda ^2+a_1\Lambda +a_0$ is a real univariate polynomial of degree 3 whose coefficients are given by

$$\begin{aligned} a_0&= w_\emptyset = 1,\\ a_1&= w_1+w_2+w_3,\\ a_2&= w_1w_2w_{12}+w_1w_3w_{13}+w_2w_3w_{23},\\ a_3&= w_1w_2w_3w_{12}w_{13}w_{23}w_{123}. \end{aligned}$$

Note that two different molecules may have the same binding polynomial and thus the same binding curve, e.g.,

$$\begin{aligned} \begin{array}{rccccccccl} &{} w_\emptyset &{} w_1 &{} w_2 &{} w_3 &{} w_{12} &{} w_{13} &{} w_{23} &{} w_{123} \\ \mathrm{W}= (&{} 1 &{} 3 &{} 1 &{} 1&{} 1&{} 1&{} 2&{} 2&{}),\\ \mathrm{W}'= (&{} 1 &{} 2 &{} 2 &{} 1&{} 1&{} 1&{} 1&{} 3&{}).\\ \end{array} \end{aligned}$$

Hence, the map $\Phi$ which maps a molecule with *n* sites to its binding polynomial is not injective.

Computing hypothetical or real molecules $\mathrm{W}$ with a fixed binding polynomial *P* can be a hard task, though there are well established algorithms and software for both. Let ${\mathcal {M}}_{\mathbb {C}}(P)$ resp. ${\mathcal {M}}_{\mathbb {R}}(P)$ denote the set of hypothetical resp. real molecules $\mathrm{W}$ with $\Phi (\mathrm{W})=P$. Then ${\mathcal {M}}_{\mathbb {C}}(P)$ is a *algebraic variety*, i.e., a set of complex points given by polynomial equations, while ${\mathcal {M}}_{\mathbb {R}}(P)$ is a *semialgebraic set*, i.e., a set of real points given by polynomial equations and inequalities.

Computing a hypothetical molecule in ${\mathcal {M}}_{\mathbb {C}}(P)$ is generally easier than ${\mathcal {M}}_{\mathbb {R}}(P)$. It can be done purely numerically, e.g., using numerical algebraic geometry and software such as bertini, PHCPack, Hom4PS or juliahomotopycontinuation. Computing a real molecule in ${\mathcal {M}}_{\mathbb {R}}(P)$ is a significantly more involved task. There are many approaches to it and most require some symbolic computation, such as RAGlib which uses a mix of symbolic and numerical computations.

However, both ${\mathcal {M}}_{\mathbb {C}}(P)$ and ${\mathcal {M}}_{\mathbb {R}}(P)$ are of high dimension and degree (Ren et al. 2019), so that it is unclear what information can be read off a randomly computed molecule. Rather, we need sensible constraints which gives us molecules of interest. Since the aim of this paper is to study cooperativity in terms of the interaction between the binding sites, we introduce the notions of absolute interaction and minimal interaction:

### Definition 2.4

The *(absolute) interaction* of a molecule $\mathrm{W}=(w_I)\in ({\mathbb {R}}_{>0})^{2^n}$ is given by

$$\begin{aligned} \Vert \mathrm{W}\Vert := \prod _{|I|>1} \max \Big (w_I,w_I^{-1}\Big ). \end{aligned}$$

Since $w_I$ represents the exponential of a difference of actual energies, which can be positive or negative, $\max (w_I,w_I^{-1})$ represents the exponential of the absolute value of that difference. Hence, the absolute interaction $\Vert \mathrm{W}\Vert$ represents the exponential of the sum of the absolute values of all energy differences. The minimal value $\Vert \mathrm{W}\Vert$ can adopt is 1 which corresponds to a minimal physical interaction energy of 0.

### Definition 2.5

Given a binding polynomial *P* of degree *n*, the *minimal interaction* is

$$\begin{aligned} \Vert P\Vert := \min \Big \{ \Vert \mathrm{W}\Vert \,\, \Big |\,\, \mathrm{W}\in ({\mathbb {R}}_{>0})^{2^n} \text { with } \Phi (\mathrm{W})=P \Big \}. \end{aligned}$$

We call a molecule *W* *minimal*, if $\Vert W\Vert = \Vert \Phi (W)\Vert$.

In other words, the minimal interaction $\Vert P\Vert$ is the minimal interaction required to generated the binding polynomial *P*. Thus it quantifies the minimal deviation from an independent system that is required to explain the observed overall binding behavior. The following proposition states that the minimum exists.

### Proposition 2.6

(Martini 2017, §4) For any univariate polynomial *P* of degree *n* with positive coefficients there exists a molecule $\mathrm{W}\in ({\mathbb {R}}_{>0})^{2^n}$ with *n* sites such that $\Phi (\mathrm{W})=P$ and $\Vert \mathrm{W}\Vert =\Vert P\Vert$.

Since cooperativity is a property of the binding curve which emerges from the interaction between the sites, the minimal interaction of a binding polynomial is a natural candidate for quantifying cooperativity. In addition, (Martini 2017, §4) shows that it has several properties that are desirable of a quantifier.

### Example 2.7

Consider the following binding polynomials:

$$\begin{aligned} P_1:=&4\Lambda ^3+3\Lambda ^2+2\Lambda +1,\\ P_2:=&6\Lambda ^3+7\Lambda ^2+4\Lambda +1=(2\Lambda +1)(3\Lambda ^2+2\Lambda +1),\\ P_3:=&6\Lambda ^3+11\Lambda ^2+6\Lambda +1=(\Lambda +1)(2\Lambda +1)(3\Lambda +1). \end{aligned}$$

A brief computation reveals that the minimal absolute interactions are

$$\begin{aligned} \Vert P_1\Vert =13.50,\quad \Vert P_2\Vert =3, \quad \Vert P_3\Vert =1. \end{aligned}$$

**Fig. 3:**
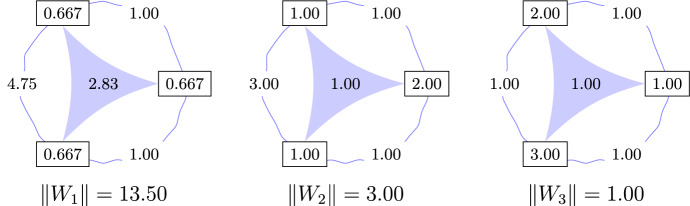
Minimal molecules for binding polynomial $P_1$, $P_2$, $P_3$

The computation for $\Vert P_1\Vert$ is explicitly shown in Example 3.3. Figure 3 illustrates the minimal molecules for each binding polynomial. Since $P_3$ factorizes into three real linear factors, its minimal molecule has only trivial interactions.

## Computing the minimal interaction

In this section, we consider the problem of computing the minimal absolute interaction for a given binding polynomial *P*:

1 $$\begin{aligned} \begin{array}{lll} \displaystyle \mathop {\mathrm{minimize}}\limits _{\mathrm{W}} &{} \quad &{} \Vert \mathrm{W}\Vert \\ \mathrm{subject~to}&{} &{} \Phi (\mathrm{W})=P \end{array} \end{aligned}$$

For the sake of clarity, we will restrict ourselves to the case of molecules with 4 binding sites, though our arguments also apply to the general case. We will discuss what makes Problem (1) challenging, and what can or cannot be done towards solving it from a practical point of view.

### The initial problem

Problem (1) is commonly called the *standard form* of a continuous optimization problem with $\Vert W\Vert$ being the *objective function* and $\Phi (\mathrm{W})=P$ the *constraints*. The set of all molecules *W* which satisfy the constraints is the *feasible set*.

Generally speaking, there are many approaches for solving optimization problems depending on the type of the objective function and the constraints, which may also include inequalities. If both are linear, the problem is also known as a *linear program*. If the objective function is quadratic and the constraints are linear, the problem is also called a *quadratic program*. Both types of optimization problems are well-studied with plenty of literature and software solutions. If both objective function and constraints are polynomial, the problem is a *polynomial optimization problem* or an *algebraic optimization problem*. This is the type of problem that needs to be solved for Problem 1, and solving it generally involves using sums of squares approaches (Lasserre 2001; Parrilo 2003) or critical point methods (Greuet and Safey El Din 2014).

For molecules with 4 binding sites specifically, Problem (1) is of the following form for a given binding polynomial $P=a_4\Lambda ^4+a_3\Lambda ^3+a_2\Lambda ^2+a_1\Lambda$:

2 $$\begin{aligned} \begin{array}{lcl} \displaystyle \mathop {\mathrm{minimize}}\limits _{(w_I)_{I\subseteq [4]}} &{}&{} \displaystyle \prod _{|I|>1} \max \Big (w_I,w_I^{-1}\Big )\\ \mathrm{subject~to}&{}&{} a_1 = w_1+w_2+w_3+w_4,\\ &{}&{} a_2 = w_1w_2w_{12}+w_1w_3w_{13}+w_1w_4w_{14}\\ &{}&{}\qquad +w_2w_3w_{23}+w_2w_4w_{24}+w_3w_4w_{34},\\ &{}&{} a_3 = w_1w_2w_3w_{12}w_{13}w_{23}w_{123}+w_1w_2w_4w_{12}w_{14}w_{24}w_{124}\\ &{}&{} \qquad +w_1w_3w_4w_{13}w_{14}w_{34}w_{134}+w_2w_3w_4w_{23}w_{24}w_{34}w_{234},\\ &{}&{} a_4 = w_1w_2w_3w_4w_{12}w_{13}w_{14}w_{23}w_{24}w_{34}w_{123}w_{124}w_{134}w_{234}w_{1234}. \end{array} \end{aligned}$$

Problem (2) is extremely hard to solve because of two reasons:

- the objective function contains 11 maxima,
- the constraints contain polynomials of degree up to 15.

In the following, we will briefly discuss the two challenges separately.

### Resolving the maxima in the objective function

The maxima in the objective function of Problem (2) can be resolved in two ways.

One standard way is to reformulate the objective function as a maximum of polynomial functions and linearize the objective function through additional constraints: Abbreviating $$\left( {\begin{array}{c}[4]\\>1\end{array}}\right) :=\{I\subseteq [4]\mid |I|>1\}$$, we define for each set of interactions $${\mathcal {I}}\subseteq \left( {\begin{array}{c}[4]\\ >1\end{array}}\right) $$

$$\begin{aligned} f_{{\mathcal {I}}}((w_I)_{I\subseteq [4]}) := \Big (\prod _{\begin{array}{c} I\in \left( {\begin{array}{c}[4]\\>1\end{array}}\right) \\ I\in {\mathcal {I}} \end{array}} w_I^{-1} \Big )\cdot \Big (\prod _{\begin{array}{c} I\in \left( {\begin{array}{c}[4]\\ >1\end{array}}\right) \\ I\not \in {\mathcal {I}} \end{array}} w_I\Big ), \end{aligned}$$

so that

$$\begin{aligned} \Vert (w_I)_{I\subseteq [4]}\Vert = \max _{{\mathcal {I}}\subseteq \left( {\begin{array}{c}[4]\\ >1\end{array}}\right) } f_{{\mathcal {I}}}((w_I)_{I\subseteq [4]}). \end{aligned}$$

Then Problem (2) can be written as:

3 $$\begin{aligned} \begin{array}{lcl} \displaystyle \mathop {\mathrm{minimize}}\limits _{(w_I)_{I\subseteq [4]}} &{}&{} r\\ \mathrm{subject~to}&{}&{} f_{{\mathcal {I}}}((w_I)_{I\subseteq [4]})\le r \quad \text {for all } {\mathcal {I}}\subseteq \left( {\begin{array}{c}[4]\\ >1\end{array}}\right) ,\\ &{}&{} a_1 = w_1+w_2+w_3+w_4,\\ &{}&{} a_2 = w_1w_2w_{12}+w_1w_3w_{13}+w_1w_4w_{14}\\ &{}&{}\qquad \quad +w_2w_3w_{23}+w_2w_4w_{24}+w_3w_4w_{34},\\ &{}&{} a_3 = w_1w_2w_3w_{12}w_{13}w_{23}w_{123}+w_1w_2w_4w_{12}w_{14}w_{24}w_{124}\\ &{}&{} \qquad \quad +w_1w_3w_4w_{13}w_{14}w_{34}w_{134}+w_2w_3w_4w_{23}w_{24}w_{34}w_{234},\\ &{}&{} a_4 = w_1w_2w_3w_4w_{12}w_{13}w_{14}w_{23}w_{24}w_{34}w_{123}w_{124}w_{134}w_{234}w_{1234}. \end{array} \end{aligned}$$

This resolves all maxima in the objective function at the cost of introducing $2^{11}$ additional constraints, one for each $${\mathcal {I}}\subseteq \left( {\begin{array}{c}[4]\\ >1\end{array}}\right) $$.

Alternatively, one can decompose the space of molecules $({\mathbb {R}}_{>0})^{2^4}$ into $2^{11}$ regions in which either $w_I\ge 1$ or $w_I\le 1$ for all $${\mathcal {I}}\subseteq \left( {\begin{array}{c}[4]\\ >1\end{array}}\right) $$. For each $${\mathcal {I}}\subseteq \left( {\begin{array}{c}[4]\\ >1\end{array}}\right) $$, we define a region

$$\begin{aligned} O_{{\mathcal {I}}}&:= \big \{(w_I)_{I \subseteq [n]} \in ({\mathbb {R}}_{>0})^{2^4}\mid \text {for all } I\in \textstyle \left( {\begin{array}{c}[4]\\ >1\end{array}}\right) :\;\; w_I\ge 1 \text { if } I\notin {\mathcal {I}} \text { and }\\&\quad w_I\le 1 \text { if } I\in {\mathcal {I}} \big \} \end{aligned}$$

so that

$$\begin{aligned} ({\mathbb {R}}_{>0})^{2^4} = \bigcup _{{\mathcal {I}}\subseteq \left( {\begin{array}{c}[4]\\ >1\end{array}}\right) } O_{{\mathcal {I}}} \quad \text {and} \quad \Vert W\Vert = f_{{\mathcal {I}}}(W) \text { for } \mathrm{W}\in {\mathcal {O}}_{{\mathcal {I}}}. \end{aligned}$$

Finding the minimal absolute interaction in $O_{{\mathcal {I}}}$ hence becomes a straight-forward polynomial optimization problem with few constraints.

4 $$\begin{aligned} \begin{array}{lll} \displaystyle \mathop {\mathrm{minimize}}\limits _{\mathrm{W}\in O_{{\mathcal {I}}}} &{} \quad &{} f_{{\mathcal {I}}}(\mathrm{W}) \\ \mathrm{subject~to}&{} &{} \Phi (\mathrm{W})=P \end{array} \end{aligned}$$

However, this approach requires solving $2^{11}$ polynomial optimization problems, one for each region ${\mathcal {O}}_{{\mathcal {I}}}$, which is not easily feasible.

One can reduce the number of regions that require consideration slightly by exploiting the intrinsic symmetry of the problem.

#### Example 3.1

Consider for example the following polynomial optimization problem:

$$\begin{aligned} \begin{array}{lcl} \displaystyle \mathop {\mathrm{minimize}}\limits _{(x,y)\in {\mathbb {R}}_{>0}^2} &{}&{} \max (x,x^{-1})\cdot \max (y,y^{-1})\\ \mathrm{subject~to}&{}&{} a_1 = x+y,\\ &{}&{} a_2 = x\cdot y. \end{array} \end{aligned}$$

**Fig. 4:**
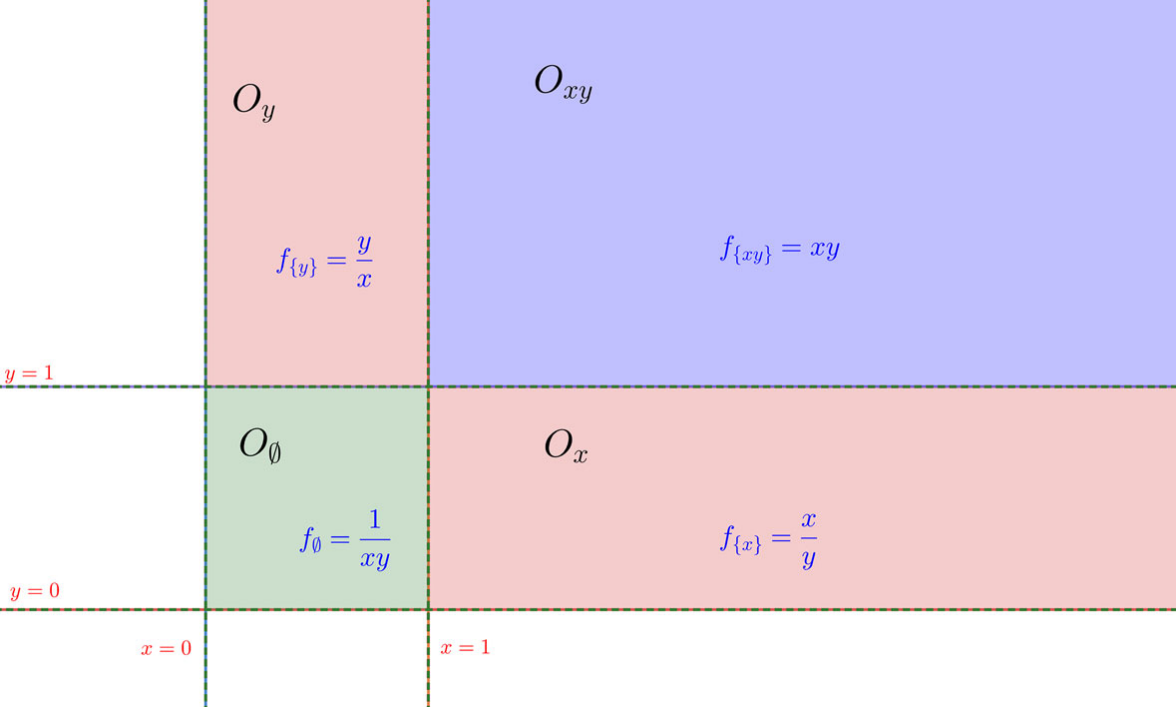
Partitions in plane

The space ${\mathbb {R}}_{>0}^2$ can be decomposed into 4 regions on which the objective function is *xy*, $\frac{x}{y}$, $\frac{y}{x}$, $\frac{1}{xy}$ respectively, see Fig. 4. As both objective function and constraints are symmetric with respect to permuting *x* and *y*, the minimum of $f_{\{y\}}$ in $O_{y}$ is same as the minimum of $f_{\{x\}}$ in $O_{x}$. Therefore, it suffices to minimize over three regions instead of all four.

For Problem (2) specifically, this means:

#### Proposition 3.2

There exists a natural action of the symmetric group $S_4$ on the space of molecules $({\mathbb {R}}_{>0})^{2^4}$ by permuting the 4 binding sites:

$$\begin{aligned} S_4\times ({\mathbb {R}}_{>0})^{2^4} \longrightarrow ({\mathbb {R}}_{>0})^{2^4},\quad (\sigma ,(w_I)) \longmapsto \sigma \cdot (w_I) := (w_{\sigma (I)}). \end{aligned}$$

This action permutes the regions ${\mathcal {O}}_{{\mathcal {I}}}$ and preserves binding polynomials as well as absolute interactions, i.e., $\Phi (\sigma \cdot \mathrm{W})=\Phi (\mathrm{W})$ and $\Vert \sigma \cdot \mathrm{W}\Vert = \Vert \mathrm{W}\Vert$ for all $\mathrm{W}\in ({\mathbb {R}}_{>0})^{2^4}$ and all $\sigma \in S_4$.

Instead of computing in all $2^{11}$ regions, it therefore suffices to consider one region per $S_4$-orbit of regions. However, this still requires 249 regions to be checked, which is still too much for practical purposes.

### Reducing the degree of the constraints

Given a binding polynomial *P*, the polynomial equations in Problem (3) arising from its coefficients $a_1,\ldots , a_4$ pose a serious computational problem. To sidestep this issue we now introduce new coordinates $s_I:=\prod _{I'\subseteq I}w_{I'}$. Here, $s_I$ represents the free energy difference of microstate *I* to the fully unoccupied state. On the positive orthant $({\mathbb {R}}_{>0})^{2^n}$, this defines a bijection


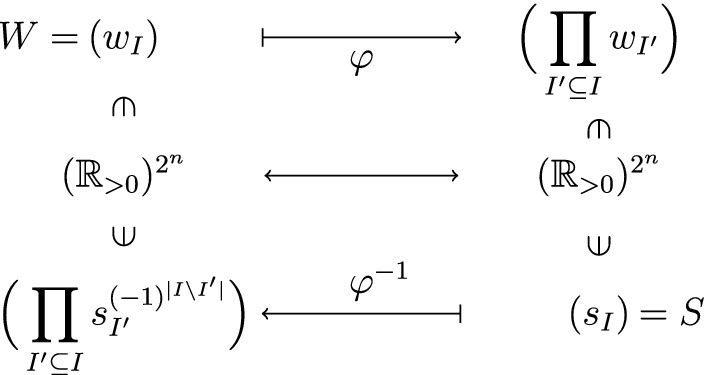


In the new coordinates, the formerly polynomial constraints $a_k = \sum _{|I|=k} \prod _{I'\subseteq I} w_{I'}$ become linear constraints $a_k=\sum _{|I|=k} s_I$ for $k=1,\ldots ,n$. While the functions $f_{{\mathcal {I}}}$ become more complicated in the new coordinates, they still remain monomial and thus it has minimal impact on the runtime: For example, for ${\mathcal {I}}=\{ \{1,2,3\} \}$

$$\begin{aligned} f_{{\mathcal {I}}} (W) = w_{123}^{-1}\cdot \prod _{\begin{array}{c} |I|>1,\\ I\ne \{1,2,3\} \end{array}} w_I \quad \text {becomes} \quad f_{{\mathcal {I}}}\circ \varphi ^{-1} (S) = \frac{s_{1234}s_{12}^2s_{13}^2s_{23}^2}{s_{123}^2s_1^3s_2^3s_3^3s_4}. \end{aligned}$$

In the new coordinates, Problem (3) becomes:

5 $$\begin{aligned} \begin{array}{lcl} \displaystyle \mathop {\mathrm{minimize}}\limits _{(w_I)_{I\subseteq [4]}} &{}&{} r\\ \mathrm{subject~to}&{}&{} f_{{\mathcal {I}}}\circ \varphi ^{-1}(S)\le r \quad \text {for all } {\mathcal {I}}\subseteq \{I\subseteq [n]\mid |I|>1\},\\ &{}&{} a_1 = s_1+s_2+s_3+s_4,\\ &{}&{} a_2 = s_{12}+s_{13}+s_{14}+s_{23}+s_{24}+s_{34},\\ &{}&{} a_3 = s_{123}+s_{124}+s_{134}+s_{234},\\ &{}&{} a_4 = s_{1234}. \end{array} \end{aligned}$$

### Solving the polynomial optimization problem

We will use SCIP (Gleixner et al. 2018) to solve polynomial optimization Problem (5), which is currently one of the fastest non-commercial solvers for non-linear programming. It uses a branch and bound method to solve the optimization problem with non-linear constraints. Example 3.3 shows a computation using SCIP for a molecule with 3 binding sites.

#### Example 3.3

**Fig. 5:**
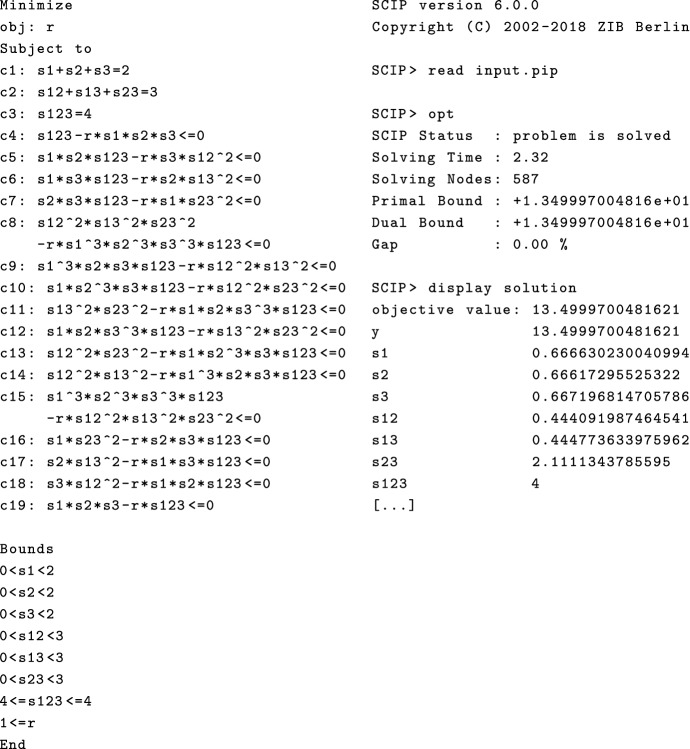
Computing the minimal absolute interaction for a molecule with 3 sites using SCIP

Consider the polynomial $P_1$ of Example 2.7. Figure 5 shows the full input on the left and the partial output on the right. In the input, the first constraints c1, c2, c3 enforce $\sum _{|I|=k} s_I= a_k$ for $i=1,2,3$. The remaining constraints c4 to c19 enforce $f_{{\mathcal {I}}}\circ \varphi ^{-1}(S)\le r$. For example, c19 states that $s_1s_2s_3 / s_{123}\le r$ which is equivalent to $f_{\emptyset }(W)=(w_{123}w_{12}w_{13}w_{23})^{-1} \le r$ in the coordinates $w_I$. The output states an approximate optimal value of $r=13.5$ and lists all values of $s_I$ of the minimal molecule, which gave rise to the numbers in Example 2.7.

Unfortunately, the $2^{11}$ constraints in Problem (5) make it practically impossible to solve directly. Instead, we focus on computing an upper bound $b_{+}$ and a lower bound $b_{-}$ for the minimal interaction. Both bounds are easier to compute, because they arise from problems with significantly less constraints. Our aim is to obtain bounds which either coincide or are sufficiently close to each other to allow us to compare the different minimal interactions with each other.

#### The upper bound $b_{+}$

Our upper bound $b_{+}$ for the solution to Problem (5) is the solution to Problem (4) for a region $O_{{\mathcal {I}}}$. In other words, instead of minimizing the interaction over all molecules in $({\mathbb {R}}_{>0})^{2^4}$, we only minimize it over the molecules in $O_{{\mathcal {I}}}$. It is an upper bound as the global minimum need not be in the region that we have chosen. Computing the solution to Problem (4) still required changing to the new coordinates $(s_I)$. Our region of choice was $O_\emptyset$, i.e., the region where $w_I\ge 1$ for all $$I\in \left( {\begin{array}{c}[4]\\ >1\end{array}}\right) $$, as it was the only region in which all computations terminated successfully during testing.

#### The lower bound $b_{-}$

Our lower bound $b_{-}$ is the solution to a relaxed Problem (5) with some of the $2^{11}$ constraints of the form $f_{{\mathcal {I}}}\circ \varphi ^{-1}(S)\le r$ removed. It is a lower bound since removing constraints enlarges the feasible set over which the minimum is computed. Appendix A contains a list of constraints that remain in the final computation. We picked a region $O_{{\mathcal {I}}}$ in each $S_4$ orbit of regions and only considered constraints given by functions $f_{{\mathcal {I}}}$ that are dominant in one of the regions.

## Experimental results

In this section, we present the results of computational experiments on data from (Imai 1973; Ikeda-Saito et al. 1983) which is also summarized in Connelly et al. (1986). Section 4.1 summarizes the origins of the data, Sect. 4.2 contains the bounds on the minimal interactions, and Sect. 4.3 contains molecules attaining the upper bound.

### Data

The first data set consists of eight oxygen binding polynomials ($P_1$ to $P_8$) of human adult hemoglobin in its native form and chemically treated with iodoacetamide, *N*-ethylmaeimide, and carboxypeptidase A. The oxygen binding was observed both in absence and in presence of 2,3-diphosphoglycerate (DPG) (Imai 1973). One of the goals was to determine how the chemical treatments and DPG affect the binding affinity of hemoglobin, the results are indicated by $\succ$ in Table 1.

**Table 1:** Conditions under which binding polynomials 1 to 8 were measured

Human Hb	In the absence of 2mM DPG		In the presence of 2mM DPG
Untreated	$P_1$	$\prec$	$P_2$
	$\curlyvee$		$\curlyvee$
Treated with iodoacetamide	$P_3$	$\prec$	$P_4$
	$\curlyvee$		$\curlyvee$
Treated with *N*-ethylmaeimide	$P_5$	$\prec$	$P_6$
	$\curlyvee$		$\curlyvee$
Treated with carboxypeptidase A	$P_7$	$\prec$	$P_8$

$P_i\prec P_j$ means $P_i$ that the Hill plot of $P_i$ has a lower maximal slope compared to $P_j$

The second data set contains five binding polynomials ($P_9$ to $P_{13}$) of native hemoglobin HbII of *Scapharca inaequivalvis* measured at different temperatures (Ikeda-Saito et al. 1983, Table 3), see Table 2. One of the goals was to investigate how temperature affects cooperativity in oxygen binding.

**Table 2:** Temperature in degree Celsius at which binding polynomials 9–13 were determined

Clam HbII	at $10{}^\circ$	at $15{}^\circ$	at $20{}^\circ$	at $25{}^\circ$	at 30${}^\circ$
	$P_{9}$	$P_{10}$	$P_{11}$	$P_{12}$	$P_{13}$

Table 3 lists the coefficients $a_1$, $a_2$ and $a_3$ of the binding polynomials $P_1$ to $P_{13}$, the coefficients $a_0$ and $a_4$ are normalized to 1. Furthermore, it lists the maximal slope to the Hill plot $n_{max}$, as reported by Imai (1973) and Ikeda-Saito et al. (1983), which relates the variance of the probability distribution on the macrostates $\{0,1,2,3,4\}$ at the respective ligand activity to the variance of a binomial distribution with the same mean (Abeliovich 2005; Martini et al. 2016a). A value larger than 1 indicates a variance higher than the maximal value an independent system can generate.

### Minimal absolute interactions

Table 3 lists the upper ($b_+$) and lower ($b_-$) bounds for the minimal absolute interaction for the binding polynomials $P_1$ to $P_{13}$. As explained in Sect. 3.4, solving Problem (5) for $n=4$ is very hard, which is why we computed bounds instead. The upper bound is the minimal absolute interaction in the region ${\mathcal {O}}_{\emptyset }$, i.e., the minimal interaction in the region where all interactions are 1 or larger. The lower bound comes from a relaxation of Problem (5) which enlarges the set of molecules over which the minimum is computed.

**Table 3:** Binding polynomials, bounds on the minimal absolute interaction and $n_{max}$ as reported by Ikeda-Saito et al. (1983), Imai (1973)

	$a_1$	$a_2$	$a_3$	$b_{+}$	$b_{-}$	$n_{max}$
$P_{1}$	0.835	0.379	0.541	527	527	2.51
$P_{2}$	0.789	0.154	0.0648	3322	1600	3.09
$P_{3}$	1.42	2.42	0.752	111	63	1.63
$P_{4}$	0.647	0.568	0.0986	2002	1460	2.71
$P_{5}$	2.0	2.31	2.04	16	16	1.44
$P_{6}$	0.539	0.909	0.554	3033	3030	2.27
$P_{7}$	3.47	4.74	2.76	2.27	1.68	1.15
$P_{8}$	3.26	5.36	2.23	7.64	2.19	1.23
$P_{9}$	1.4	1.0	0.62	66	66	2.08
$P_{10}$	1.4	0.96	0.60	66	66	2.10
$P_{11}$	1.2	0.93	0.70	123	123	2.08
$P_{12}$	1.4	0.95	0.62	66	66	2.10
$P_{13}$	1.1	0.98	0.59	175	175	2.12

Coefficients $a_0$ and $a_4$ are normalized to 1 for all polynomials

Looking at the bounds $b_+$ and $b_-$ in Table 3, we observe two things:

1. Apart from $P_2$, $P_3$, and $P_8$, the bounds determine the minimal interaction up to a precision of $15\%$.
2. The bounds are almost sufficient to rank the minimal interactions of $P_1$ to $P_8$ and $P_9$ to $P_{13}$:
  $$\begin{aligned}&\Vert P_6\Vert> \Vert P_4\Vert> \Vert P_1\Vert> \Vert P_3\Vert> \Vert P_5\Vert> \Vert P_7\Vert> \Vert P_8\Vert \\&\quad \text {and}\qquad \Vert P_9\Vert = \Vert P_{10}\Vert = \Vert P_{12}\Vert> \Vert P_{11}\Vert > \Vert P_{13}\Vert \end{aligned}$$
  with $\Vert P_2\Vert$ possibly being larger than $\Vert P_6\Vert$ or between $\Vert P_4\Vert$ and $\Vert P_1\Vert$.

Comparing the ranking of the binding polynomials by their minimal interactions with the ranking by the maximal slope of their Hill plot $n_{\max }$ reveals the following similarities and differences:

#### $P_9$ to $P_{13}$

**Fig. 6:**
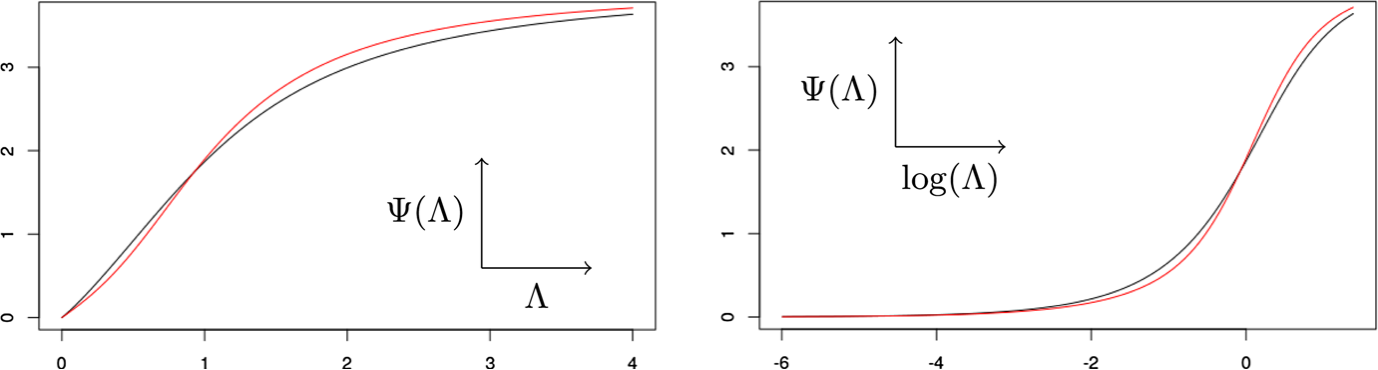
The binding curves of $P_9$ (black) and $P_{11}$ (red) in normal (left) and logarithmic (right) scale (color figure online)

We begin by looking at polynomials $P_9$ to $P_{13}$ as there are prominent differences between minimal interactions and maximal slopes of the Hill plots: While all maximal slopes are approximately equal, there are stark differences in the minimal interactions. This is because the minimal interaction depends on the entire binding curve, while the maximal slope does not. Figure 6 illustrates that while the binding curves of $P_9$ to $P_{13}$ may have identical maximal slopes in logarithmic coordinates, there are subtle differences between both curves. The binding curve of $P_{11}$ has a stronger sigmoidal shape, which is why more interaction is necessary to generate the binding behaviour.

#### $P_1$ to $P_8$

**Fig. 7:**
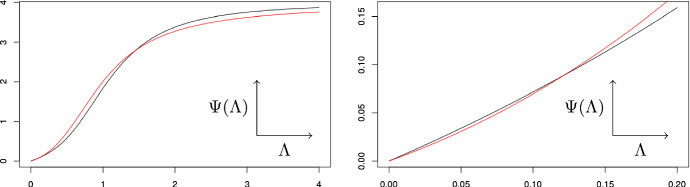
The binding curves of $P_4$ (black) and $P_6$ (red) on [0, 4] (left) and zoomed in on [0, 0.2] (right). Note how the red curve starts and ends lower as the black curve, but overtakes it in between (color figure online)

While the ranking by minimal interaction is different to the ranking by maximal slope, we retain nearly all important relations in Table 1, which indicate how the chemicals affect the binding behaviour. The only exceptions are $P_4$ and $P_6$, where $P_4$ has a higher maximal slope and $P_6$ has a higher minimal interaction. This is not surprising, as Fig. 7 illustrates that the binding curve $P_4$ has a higher maximal slope, while the binding curve $P_6$ has a stronger curvature.

Another explanation can be found in the coefficients of $P_4$ and $P_6$: In $P_6$, coefficient $a_2$ is larger than $a_1$ and $a_3$ and almost same size as $a_4=1$. This means that the distribution on the macrostates stabilizes around macrostate 2. This binding behaviour does not only requires more interaction between the binding sites, it also prevents large variances of the distribution on the macrostates and thus leads to a reduction of $n_{max}$. In $P_4$, coefficients $a_1, a_2, a_3$ are smaller than $a_4=1$. This means that the distribution on the macrostates leans toward macrostate 4, which leads to a higher value of $n_{max}$.

### Molecules solving the optimization problem

Table 4 lists all molecules realizing the upper bound $b_{+}$ of the minimal interaction. We see that the many interaction energies are close to 1. This is not surprising since we are minimizing the exponential of an $L_1$-norm and $L_1$ optimizations are known to provide sparse solutions (Tibshirani 1996).

Looking at the structure of the solutions in more detail, we see that most solutions include a non-trivial weight for a pairwise interaction ($w_{34}$). The only polynomial whose molecule possesses trivial pairwise interaction is $P_2$ which has the highest $n_{max}$. This is plausible as a high $n_{max}$ means a high variance of the macroscopic distribution, which in turn implies more weights towards macrostates 0 and 4. And any nontrivial pairwise interaction would put more weight on macrostate 2.

Moreover, we mentioned how the macroscopic distribution for $P_6$ stabilizes around 2 while the macroscopic distribution for $P_4$ leans towards 4. This can be seen in their minimal molecules in $O_\emptyset$, as the molecule for $P_6$ has large $w_{34}$ and the molecule for $P_4$ has a large $w_{1234}$.

**Table 4:** Molecules of $P_1$ to $P_{13}$ realizing the upper bound $b_+$

	$w_1$	$w_2$	$w_3$	$w_{4}$	$w_{12}\cdots w_{24}$	$w_{34}$	$w_{123}$	$w_{124}$	$w_{134}$	$w_{234}$	$w_{1234}$
$P_{1}$	0.209	0.209	0.209	0.209	1.00	**3.70**	1.00	1.00	**14.5**	1.00	**9.79**
$P_{2}$	0.0822	0.0822	0.0822	0.543	1.00	1.00	**6.60**	1.00	**14.7**	1.00	**34.3**
$P_{3}$	0.179	0.179	0.531	0.531	1.00	**7.12**	1.00	1.00	1.00	1.00	**15.6**
$P_{4}$	0.101	0.101	0.223	0.223	1.00	**9.42**	1.00	1.00	1.00	1.00	**212**
$P_{5}$	0.500	0.500	0.500	0.500	1.00	**4.24**	1.00	1.00	**1.69**	**1.69**	**1.32**
$P_{6}$	0.135	0.135	0.135	0.135	1.00	**45.1**	1.00	1.00	**2.49**	**2.49**	**10.9**
$P_{7}$	0.569	0.569	1.17	1.17	1.00	**1.30**	1.00	1.00	1.00	1.00	**1.75**
$P_{8}$	0.265	0.265	1.36	1.36	1.00	**2.06**	1.00	1.00	1.00	1.00	**3.70**
$P_{9}$	0.350	0.350	0.350	0.350	1.00	**3.16**	1.00	1.00	**1.97**	**1.97**	**5.43**
$P_{10}$	0.350	0.350	0.350	0.350	1.00	**2.84**	1.00	1.00	**2.11**	**2.11**	**5.26**
$P_{11}$	0.300	0.300	0.300	0.300	1.00	**5.33**	1.00	1.00	**2.24**	**2.24**	**4.60**
$P_{12}$	0.350	0.350	0.350	0.300	1.00	**2.76**	1.00	1.00	**2.26**	**2.26**	**4.73**
$P_{13}$	0.275	0.275	0.275	0.275	1.00	**7.96**	1.00	1.00	**1.66**	**1.66**	**8.01**

All interactions strictly larger than 1 are highlighted in bold

## Discussion and outlook

### Cooperativity and minimal absolute interaction

While cooperativity has always been a property of the macroscopic binding behavior, it is often interpreted on a microscopic level, for instance that “binding of a ligand molecule increases the receptor’s apparent affinity, and hence increases the chance of another ligand molecule binding” (Stefan and Le Novère 2013). There is however no straightforward connection between the macroscopic and microscopic properties, as generally there are infinitely many different molecules with the same binding curve. Thus creating a bridge between macroscopic and microscopic properties usually relies on additional restrictions, such as having indistinguishable sites.

In this paper, we considered the minimal interaction necessary to generate a fixed overall binding curve. In other words, we study the minimal deviation from a molecule with no interaction that is needed to create a prescribed binding behavior. We showed that computing the minimal interaction gives rise to a challenging polynomial optimization problem, and proposed easier ways for obtaining upper and lower bounds. Applying our techniques to overall binding curves and comparing our results to the maximal slope of the Hill plot, we observe:

1. The upper and lower bounds were sufficient to rank all but one of the curves.
2. The complete ranking of the curves by minimal interaction may differ from the ranking by maximal Hill slope, but most of the important relations are the same.
3. In the cases were minimal interaction and maximal Hill slope disagreed, the curve with the higher minimal interaction displayed a stronger curvature (see Figs. 6 and 7).

Conceptually, the minimal interaction considers the entire curve and captures any deviation from an independent system, while the maximal Hill slope only indicates an abnormally high variance of the macroscopic distributions. This is also reflected in our computations, as our results based on the minimal interaction largely coincides with the results based on the maximal Hill slope and the small differences in the results could be explained by signs of interaction outside the region of maximal Hill slope. Additionally, the corresponding minimal molecules may give valuable insights into the microscopic dependencies of the molecule. While the minimal molecule may not be a physically correct representation of the actual chemical molecule that was measured, in a way it describes the simplest possible set of interactions required for the binding curve. Note that we chose to minimize the $L_1$-norm of the actual interaction energies because it was the most natural norm in our framework. Alternatively, other $L_p$-norms could be used, and it is unclear whether they yield better qualitative results. However, with current methods the minimal interaction is much harder to compute than the maximal Hill slope and thus new mathematical approaches are required.

### The mathematical problem

Ligand binding raises many intriguing questions in applied algebra and geometry, and stands to benefit equally from further insights into the intrinsic geometry of the problems. While previous works have explored the application of polynomial system solving techniques to find decoupled molecules in a situation with different types of ligands (Ren et al. 2019; Martini et al. 2013), this work explored the use of algebraic optimization to calculate the minimal interaction required to generate a certain binding behavior.

We illustrated how searching for the minimal interaction energy required to generate a binding curve leads to a problem in algebraic optimization. Even though, computational tools are readily available and the structure of the problem seems relatively simple on first sight, it becomes complicated when more than 3 binding sites are considered. In particular the fact that we had to calculate upper and lower bounds by a relaxed problem and were not able to close their gap completely, shows that our approach is not yet ready for a simple ready-to-use tool to quantify cooperativity. Even though we were able to rank the polynomials in Sect. 4.2, we were not able to compute precise minimal energies for all polynomials. We thus believe further research into the computational problem is warranted, though it may need to come from a different direction.

#### Exploiting symmetries

One possibility would be to show that the minimal molecule have some symmetry, which would reduce the number of variables (interaction energies) in our problem. While both Problem 5 and the molecules in Table 4 show some kind of symmetry, it is mathematically not clear whether that always needs to be the case. There are results using *representation theory* which relate symmetries in polynomial optimization problems to symmetries in their solutions (Riener and Safey El Din 2018), however the assumptions on the symmetry in the polynomial optimization problem are quite strong and not satisfied by Problem 5.

#### Tropical geometry

Tropical geometry (Maclagan and Sturmfels 2015) revolves around functions on the max-plus semiring, such as the absolute interaction in logarithmic coordinates:


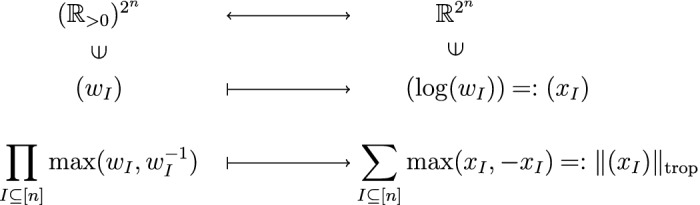


In tropical geometry, the function $\Vert \cdot \Vert _{\text {trop}}$ is called a tropical rational function. It is a piecewise-linear function, linear on the image of each ${\mathcal {O}}_{{\mathcal {I}}}$, and the boundary separating its regions of linearity is called its tropical hypersurface *T*. Since the gradient of $\Vert \cdot \Vert _{\text {trop}}$ is constant on each region of linearity, optimizing $\Vert \cdot \Vert _{\text {trop}}$ on any set *L* boils down to understanding the intersection of *L* with *T*.

For our problem, *L* is the image of the feasible set given by the conditions $\Phi (W)=P$ which is an exponential variety of codimension *n*. Theoretically, there exist only a finite number of intersection patterns of *L* and *T*. Classifying these intersection patterns would allow us to compute minimal interactions and minimal molecules almost instantly.

#### Tensors

Tensors (Landsberg 2012) are multidimensional arrays of real numbers which generalize matrices. We can regard molecules as (positive) tensors in ${\mathbb {R}}^{2\times \dots \times 2} =: ({\mathbb {R}}^2)^{\otimes n}$, whose entries are indexed by $\{0,1\}^n$ which is in bijection with the subsets of [*n*].

From this point of view, many concepts in tensor theory have natural analogues in ligand binding, such as symmetric tensors and molecules with indistinguishable sites. Notably, a rank one tensors is up to scaling a tensor with entries

$$\begin{aligned} (1,0,\dots )\!: s_1, \quad (0,1,0,\dots )\!: s_2, \quad (1,1,0,\dots )\!: s_1s_2, \quad \ldots \quad \text {for some } s_i\in {\mathbb {R}}. \end{aligned}$$

Hence rank one tensors correspond to molecules with independent sites. Therefore, decomposing a given tensor into rank one tensors translates to decomposing a given molecule into different molecules with independent sites whose mix has the same binding curve. And the tensor rank translates to the minimal number of different molecules with independent sites needed to recreate the same binding curve. Thus the tensor rank of a molecule could serve as an interesting, discrete measure for cooperativity.

#### Dependency measures

From a stochastic or statistical point of view, the problem of cooperativity is a problem of quantifying (minimal) dependency, which is a classical research topic in these fields (Rényi 1959; Schweizer and Wolff 1981; Koyak 1987). In particular copulas (Schweizer 1991; Durante and Sempi 2010; Nelsen 2007) have become a common tool to describe dependencies between random variables. Copulas cannot be applied directly to our problem since they are defined on continuous, not discrete, random variables. Moreover, they aim at modeling the dependency of given random variables which in our scenario corresponds to the dependency of the sites of a known molecule. A copula then relates the one-dimensional marginal distributions to the multivariate minimization problem of finding the minimal dependency required to generate a given distribution of a sum of dependent variables is usually not treated. Nevertheless, the problem of cooperativity should also be treated from a more probabilistic point of view and the available concepts should be considered in more detail in the context of ligand binding.

## Appendix A. List of constraints

Below is a compact representation of the list of 180 constraints we considered to compute the lower bounds $b_{-}$ of minimal absolute interaction in Fig. 3. There, $\{12,34,123\}$ represents the constraint $f_{{\mathcal {I}}}\le r$ for ${\mathcal {I}}=\{12,34,123\}$, which is the absolute interaction $\Vert W\Vert$ for $W\in {\mathcal {O}}_{{\mathcal {I}}}$ where $w_{12},w_{34},w_{123}\le 1$ and $w_{I} \ge 1$ otherwise.

$$\emptyset $$, $$\{12\}$$, $$\{123\}$$, $$\{1234\}$$, $$\{12$$, $$13\}$$, $$\{12$$, $$34\}$$, $$\{12$$, $$123\}$$, $$\{12$$, $$134\}$$, $$\{123$$, $$124\}$$, $$\{12$$, $$1234\}$$, $$\{123$$, $$1234\}$$, $$\{12$$, $$13$$, $$14\}$$, $$\{12$$, $$13$$, $$23\}$$, $$\{12$$, $$13$$, $$24\}$$, $$\{12$$, $$13$$, $$123\}$$, $$\{12$$, $$13$$, $$124\}$$, $$\{12$$, $$34$$, $$123\}$$, $$\{12$$, $$13$$, $$234\}$$, $$\{12$$, $$123$$, $$124\}$$, $$\{12$$, $$123$$, $$134\}$$, $$\{12$$, $$134$$, $$234\}$$, $$\{123$$, $$124$$, $$134\}$$, $$\{12$$, $$13$$, $$1234\}$$, $$\{12$$, $$34$$, $$1234\}$$, $$\{12$$, $$123$$, $$1234\}$$, $$\{12$$, $$134$$, $$1234\}$$, $$\{123$$, $$124$$, $$1234\}$$, $$\{12$$, $$13$$, $$14$$, $$23\}$$, $$\{12$$, $$13$$, $$24$$, $$34\}$$, $$\{12$$, $$13$$, $$14$$, $$123\}$$, $$\{12$$, $$13$$, $$23$$, $$123\}$$, $$\{12$$, $$13$$, $$24$$, $$123\}$$, $$\{12$$, $$13$$, $$23$$, $$124\}$$, $$\{12$$, $$13$$, $$24$$, $$134\}$$, $$\{12$$, $$13$$, $$14$$, $$234\}$$, $$\{12$$, $$13$$, $$123$$, $$124\}$$, $$\{12$$, $$13$$, $$124$$, $$134\}$$, $$\{12$$, $$13$$, $$123$$, $$234\}$$, $$\{12$$, $$34$$, $$123$$, $$134\}$$, $$\{12$$, $$34$$, $$123$$, $$124\}$$, $$\{12$$, $$13$$, $$124$$, $$234\}$$, $$\{12$$, $$123$$, $$124$$, $$134\}$$, $$\{12$$, $$123$$, $$134$$, $$234\}$$, $$\{123$$, $$124$$, $$134$$, $$234\}$$, $$\{12$$, $$13$$, $$14$$, $$1234\}$$, $$\{12$$, $$13$$, $$23$$, $$1234\}$$, $$\{12$$, $$13$$, $$24$$, $$1234\}$$, $$\{12$$, $$13$$, $$123$$, $$1234\}$$, $$\{12$$, $$13$$, $$124$$, $$1234\}$$, $$\{12$$, $$34$$, $$123$$, $$1234\}$$, $$\{12$$, $$13$$, $$234$$, $$1234\}$$, $$\{12$$, $$123$$, $$124$$, $$1234\}$$, $$\{12$$, $$123$$, $$134$$, $$1234\}$$, $$\{12$$, $$134$$, $$234$$, $$1234\}$$, $$\{123$$, $$124$$, $$134$$, $$1234\}$$, $$\{12$$, $$13$$, $$14$$, $$23$$, $$24\}$$, $$\{12$$, $$13$$, $$14$$, $$23$$, $$123\}$$, $$\{12$$, $$13$$, $$14$$, $$23$$, $$124\}$$, $$\{12$$, $$13$$, $$24$$, $$34$$, $$123\}$$, $$\{12$$, $$13$$, $$14$$, $$23$$, $$234\}$$, $$\{12$$, $$13$$, $$14$$, $$123$$, $$124\}$$, $$\{12$$, $$13$$, $$23$$, $$123$$, $$124\}$$, $$\{12$$, $$13$$, $$24$$, $$123$$, $$124\}$$, $$\{12$$, $$13$$, $$24$$, $$123$$, $$234\}$$, $$\{12$$, $$13$$, $$24$$, $$123$$, $$134\}$$, $$\{12$$, $$13$$, $$23$$, $$124$$, $$134\}$$, $$\{12$$, $$13$$, $$14$$, $$123$$, $$234\}$$, $$\{12$$, $$13$$, $$24$$, $$134$$, $$234\}$$, $$\{12$$, $$13$$, $$123$$, $$124$$, $$134\}$$, $$\{12$$, $$13$$, $$123$$, $$124$$, $$234\}$$, $$\{12$$, $$34$$, $$123$$, $$124$$, $$134\}$$, $$\{12$$, $$13$$, $$124$$, $$134$$, $$234\}$$, $$\{12$$, $$123$$, $$124$$, $$134$$, $$234\}$$, $$\{12$$, $$13$$, $$14$$, $$23$$, $$1234\}$$, $$\{12$$, $$13$$, $$24$$, $$34$$, $$1234\}$$, $$\{12$$, $$13$$, $$14$$, $$123$$, $$1234\}$$, $$\{12$$, $$13$$, $$23$$, $$123$$, $$1234\}$$, $$\{12$$, $$13$$, $$24$$, $$123$$, $$1234\}$$, $$\{12$$, $$13$$, $$23$$, $$124$$, $$1234\}$$, $$\{12$$, $$13$$, $$24$$, $$134$$, $$1234\}$$, $$\{12$$, $$13$$, $$14$$, $$234$$, $$1234\}$$, $$\{12$$, $$13$$, $$123$$, $$124$$, $$1234\}$$, $$\{12$$, $$13$$, $$124$$, $$134$$, $$1234\}$$, $$\{12$$, $$13$$, $$123$$, $$234$$, $$1234\}$$, $$\{12$$, $$34$$, $$123$$, $$134$$, $$1234\}$$, $$\{12$$, $$34$$, $$123$$, $$124$$, $$1234\}$$, $$\{12$$, $$13$$, $$124$$, $$234$$, $$1234\}$$, $$\{12$$, $$123$$, $$124$$, $$134$$, $$1234\}$$, $$\{12$$, $$123$$, $$134$$, $$234$$, $$1234\}$$, $$\{123$$, $$124$$, $$134$$, $$234$$, $$1234\}$$, $$\{12$$, $$13$$, $$14$$, $$23$$, $$24$$, $$34\}$$, $$\{12$$, $$13$$, $$14$$, $$23$$, $$24$$, $$123\}$$, $$\{12$$, $$13$$, $$14$$, $$23$$, $$24$$, $$134\}$$, $$\{12$$, $$13$$, $$14$$, $$23$$, $$123$$, $$124\}$$, $$\{12$$, $$13$$, $$24$$, $$34$$, $$123$$, $$234\}$$, $$\{12$$, $$13$$, $$14$$, $$23$$, $$124$$, $$134\}$$, $$\{12$$, $$13$$, $$14$$, $$23$$, $$123$$, $$234\}$$, $$\{12$$, $$13$$, $$24$$, $$34$$, $$123$$, $$124\}$$, $$\{12$$, $$13$$, $$14$$, $$23$$, $$124$$, $$234\}$$, $$\{12$$, $$13$$, $$14$$, $$123$$, $$124$$, $$134\}$$, $$\{12$$, $$13$$, $$23$$, $$123$$, $$124$$, $$134\}$$, $$\{12$$, $$13$$, $$24$$, $$123$$, $$124$$, $$134\}$$, $$\{12$$, $$13$$, $$14$$, $$123$$, $$124$$, $$234\}$$, $$\{12$$, $$13$$, $$24$$, $$123$$, $$134$$, $$234\}$$, $$\{12$$, $$13$$, $$23$$, $$124$$, $$134$$, $$234\}$$, $$\{12$$, $$13$$, $$123$$, $$124$$, $$134$$, $$234\}$$, $$\{12$$, $$34$$, $$123$$, $$124$$, $$134$$, $$234\}$$, $$\{12$$, $$13$$, $$14$$, $$23$$, $$24$$, $$1234\}$$, $$\{12$$, $$13$$, $$14$$, $$23$$, $$123$$, $$1234\}$$, $$\{12$$, $$13$$, $$14$$, $$23$$, $$124$$, $$1234\}$$, $$\{12$$, $$13$$, $$24$$, $$34$$, $$123$$, $$1234\}$$, $$\{12$$, $$13$$, $$14$$, $$23$$, $$234$$, $$1234\}$$, $$\{12$$, $$13$$, $$14$$, $$123$$, $$124$$, $$1234\}$$, $$\{12$$, $$13$$, $$23$$, $$123$$, $$124$$, $$1234\}$$, $$\{12$$, $$13$$, $$24$$, $$123$$, $$124$$, $$1234\}$$, $$\{12$$, $$13$$, $$24$$, $$123$$, $$234$$, $$1234\}$$, $$\{12$$, $$13$$, $$24$$, $$123$$, $$134$$, $$1234\}$$, $$\{12$$, $$13$$, $$23$$, $$124$$, $$134$$, $$1234\}$$, $$\{12$$, $$13$$, $$14$$, $$123$$, $$234$$, $$1234\}$$, $$\{12$$, $$13$$, $$24$$, $$134$$, $$234$$, $$1234\}$$, $$\{12$$, $$13$$, $$123$$, $$124$$, $$134$$, $$1234\}$$, $$\{12$$, $$13$$, $$123$$, $$124$$, $$234$$, $$1234\}$$, $$\{12$$, $$34$$, $$123$$, $$124$$, $$134$$, $$1234\}$$, $$\{12$$, $$13$$, $$124$$, $$134$$, $$234$$, $$1234\}$$, $$\{12$$, $$123$$, $$124$$, $$134$$, $$234$$, $$1234\}$$, $$\{12$$, $$13$$, $$14$$, $$23$$, $$24$$, $$34$$, $$123\}$$, $$\{12$$, $$13$$, $$14$$, $$23$$, $$24$$, $$123$$, $$124\}$$, $$\{12$$, $$13$$, $$14$$, $$23$$, $$24$$, $$123$$, $$134\}$$, $$\{12$$, $$13$$, $$14$$, $$23$$, $$24$$, $$134$$, $$234\}$$, $$\{12$$, $$13$$, $$14$$, $$23$$, $$123$$, $$124$$, $$134\}$$, $$\{12$$, $$13$$, $$14$$, $$23$$, $$123$$, $$124$$, $$234\}$$, $$\{12$$, $$13$$, $$24$$, $$34$$, $$123$$, $$124$$, $$134\}$$, $$\{12$$, $$13$$, $$14$$, $$23$$, $$124$$, $$134$$, $$234\}$$, $$\{12$$, $$13$$, $$14$$, $$123$$, $$124$$, $$134$$, $$234\}$$, $$\{12$$, $$13$$, $$23$$, $$123$$, $$124$$, $$134$$, $$234\}$$, $$\{12$$, $$13$$, $$24$$, $$123$$, $$124$$, $$134$$, $$234\}$$, $$\{12$$, $$13$$, $$14$$, $$23$$, $$24$$, $$34$$, $$1234\}$$, $$\{12$$, $$13$$, $$14$$, $$23$$, $$24$$, $$123$$, $$1234\}$$, $$\{12$$, $$13$$, $$14$$, $$23$$, $$24$$, $$134$$, $$1234\}$$, $$\{12$$, $$13$$, $$14$$, $$23$$, $$123$$, $$124$$, $$1234\}$$, $$\{12$$, $$13$$, $$24$$, $$34$$, $$123$$, $$234$$, $$1234\}$$, $$\{12$$, $$13$$, $$14$$, $$23$$, $$124$$, $$134$$, $$1234\}$$, $$\{12$$, $$13$$, $$14$$, $$23$$, $$123$$, $$234$$, $$1234\}$$, $$\{12$$, $$13$$, $$24$$, $$34$$, $$123$$, $$124$$, $$1234\}$$, $$\{12$$, $$13$$, $$14$$, $$23$$, $$124$$, $$234$$, $$1234\}$$, $$\{12$$, $$13$$, $$14$$, $$123$$, $$124$$, $$134$$, $$1234\}$$, $$\{12$$, $$13$$, $$23$$, $$123$$, $$124$$, $$134$$, $$1234\}$$, $$\{12$$, $$13$$, $$24$$, $$123$$, $$124$$, $$134$$, $$1234\}$$, $$\{12$$, $$13$$, $$14$$, $$123$$, $$124$$, $$234$$, $$1234\}$$, $$\{12$$, $$13$$, $$24$$, $$123$$, $$134$$, $$234$$, $$1234\}$$, $$\{12$$, $$13$$, $$23$$, $$124$$, $$134$$, $$234$$, $$1234\}$$, $$\{12$$, $$13$$, $$123$$, $$124$$, $$134$$, $$234$$, $$1234\}$$, $$\{12$$, $$34$$, $$123$$, $$124$$, $$134$$, $$234$$, $$1234\}$$, $$\{12$$, $$13$$, $$14$$, $$23$$, $$24$$, $$34$$, $$123$$, $$124\}$$, $$\{12$$, $$13$$, $$14$$, $$23$$, $$24$$, $$123$$, $$124$$, $$134\}$$, $$\{12$$, $$13$$, $$14$$, $$23$$, $$24$$, $$123$$, $$134$$, $$234\}$$, $$\{12$$, $$13$$, $$14$$, $$23$$, $$123$$, $$124$$, $$134$$, $$234\}$$, $$\{12$$, $$13$$, $$24$$, $$34$$, $$123$$, $$124$$, $$134$$, $$234\}$$, $$\{12$$, $$13$$, $$14$$, $$23$$, $$24$$, $$34$$, $$123$$, $$1234\}$$, $$\{12$$, $$13$$, $$14$$, $$23$$, $$24$$, $$123$$, $$124$$, $$1234\}$$, $$\{12$$, $$13$$, $$14$$, $$23$$, $$24$$, $$123$$, $$134$$, $$1234\}$$, $$\{12$$, $$13$$, $$14$$, $$23$$, $$24$$, $$134$$, $$234$$, $$1234\}$$, $$\{12$$, $$13$$, $$14$$, $$23$$, $$123$$, $$124$$, $$134$$, $$1234\}$$, $$\{12$$, $$13$$, $$14$$, $$23$$, $$123$$, $$124$$, $$234$$, $$1234\}$$, $$\{12$$, $$13$$, $$24$$, $$34$$, $$123$$, $$124$$, $$134$$, $$1234\}$$, $$\{12$$, $$13$$, $$14$$, $$23$$, $$124$$, $$134$$, $$234$$, $$1234\}$$, $$\{12$$, $$13$$, $$14$$, $$123$$, $$124$$, $$134$$, $$234$$, $$1234\}$$, $$\{12$$, $$13$$, $$23$$, $$123$$, $$124$$, $$134$$, $$234$$, $$1234\}$$, $$\{12$$, $$13$$, $$24$$, $$123$$, $$124$$, $$134$$, $$234$$, $$1234\}$$, $$\{12$$, $$13$$, $$14$$, $$23$$, $$24$$, $$34$$, $$123$$, $$124$$, $$134\}$$, $$\{12$$, $$13$$, $$14$$, $$23$$, $$24$$, $$123$$, $$124$$, $$134$$, $$234\}$$, $$\{12$$, $$13$$, $$14$$, $$23$$, $$24$$, $$34$$, $$123$$, $$124$$, $$1234\}$$, $$\{12$$, $$13$$, $$14$$, $$23$$, $$24$$, $$123$$, $$124$$, $$134$$, $$1234\}$$, $$\{12$$, $$13$$, $$14$$, $$23$$, $$24$$, $$123$$, $$134$$, $$234$$, $$1234\}$$, $$\{12$$, $$13$$, $$14$$, $$23$$, $$123$$, $$124$$, $$134$$, $$234$$, $$1234\}$$, $$\{12$$, $$13$$, $$24$$, $$34$$, $$123$$, $$124$$, $$134$$, $$234$$, $$1234\}$$, $$\{12$$, $$13$$, $$14$$, $$23$$, $$24$$, $$34$$, $$123$$, $$124$$, $$134$$, $$234\}$$, $$\{12$$, $$13$$, $$14$$, $$23$$, $$24$$, $$34$$, $$123$$, $$124$$, $$134$$, $$1234\}$$, $$\{12$$, $$13$$, $$14$$, $$23$$, $$24$$, $$123$$, $$124$$, $$134$$, $$234$$, $$1234\}$$, $$\{12$$, $$13$$, $$14$$, $$23$$, $$24$$, $$34$$, $$123$$, $$124$$, $$134$$, $$234$$, $$1234\}$$
